# Case report: Challenges in immune reconstitution following hematopoietic stem cell transplantation for CTLA-4 insufficiency-like primary immune regulatory disorders

**DOI:** 10.3389/fimmu.2022.1070068

**Published:** 2022-12-27

**Authors:** Adriana Margarit-Soler, Àngela Deyà-Martínez, Juan Torres Canizales, Alexandru Vlagea, Ana García-García, Júlia Marsal, Maria Trabazo Del Castillo, Sílvia Planas, Sílvia Simó, Ana Esteve-Sole, María Suárez-Lledó Grande, Isabel Badell, Montserrat Rovira Tarrats, Francesc Fernández-Avilés, Laia Alsina

**Affiliations:** ^1^ Bone Marrow Transplant Unit, Oncology Service, Hospital Sant Joan de Déu, Barcelona, Spain; ^2^ Clinical Immunology and Primary Immunodeficiencies Unit, Pediatric Allergy and Clinical Immunology Department, Hospital Sant Joan de Déu, Barcelona, Spain; ^3^ Study Group for Immune Dysfunction Diseases in Children (GEMDIP), Institut de Recerca Sant Joan de Déu, Barcelona, Spain; ^4^ Clinical Immunology Program Hospital Sant Joan de Déu-Hospital Clínic Barcelona, Barcelona, Spain; ^5^ Clinical Immunology Unit, Department of Immunology, Biomedical Diagnostic Center, Hospital Clínic of Barcelona-Institut d'Investigacions Biomèdiques August Pi i Sunyer (IDIBAPS), Barcelona, Spain; ^6^ Department of Pathology, Hospital Sant Joan de Déu, Barcelona, Spain; ^7^ Infectious Diseases Unit, Department of Pediatrics, Hospital Sant Joan de Déu, Barcelona, Spain; ^8^ Center for Biomedical Network Research on Epidemiology and Public Health (CIBERESP), Madrid, Spain; ^9^ Hematopoietic Transplantation Unit, Hematology Department, Clinical Institute of Hematology and Oncology (ICMHO), Hospital Clínic de Barcelona, Barcelona, Spain; ^10^ Institut d’Investigacions Biomèdiques August Pi i Sunyer (IDIBAPS), Hospital Clinic, Barcelona, Spain; ^11^ Department of Surgery and Surgical Specializations, Facultat de Medicina i Ciències de la Salut, Universitat de Barcelona, Barcelona, Spain; ^12^ Pediatric Haematology and Stem Cell Transplantation Unit, Pediatric Department, Hospital de la Santa Creu i Sant Pau, Universitat Autònoma de Barcelona, Barcelona, Spain

**Keywords:** CTLA-4, primary immunodeficiency, hematopoietic stem cell transplantation, abatacept, immune reconstitution, chimerism

## Abstract

Cytotoxic T-lymphocyte antigen-4 (CTLA-4) haploinsufficiency is a T-cell hyperactivation disorder that can manifest with both immunodeficiency and immune dysregulation. Approximately one-third of patients may present mild symptoms and remain stable under supportive care. The remaining patients may develop severe multiorgan autoimmunity requiring lifelong immunosuppressive treatment. Hematopoietic stem cell transplantation (HSCT) is potentially curable for patients with treatment-resistant immune dysregulation. Nevertheless, little experience is reported regarding the management of complications post-HSCT. We present case 1 (CTLA-4 haploinsufficiency) and case 2 (CTLA-4 insufficiency-like phenotype) manifesting with severe autoimmunity including cytopenia and involvement of the central nervous system (CNS), lung, and gut and variable impairment of humoral responses. Both patients underwent HSCT for which the main complications were persistent mixed chimerism, infections, and immune-mediated complications [graft-versus-host disease (GVHD) and nodular lung disease]. Detailed management and outcomes of therapeutic interventions post-HSCT are discussed. Concretely, post-HSCT abatacept and human leukocyte antigen (HLA)-matched sibling donor lymphocyte infusions may be used to increase T-cell donor chimerism with the aim of correcting the immune phenotype of CTLA-4 haploinsufficiency.

## Introduction

Regulatory T cell (Treg) defects are conditions included within the category of primary immune regulatory disorders (PIRDs) (1). They are defined by quantitative or qualitative impairment of the Treg compartment, predisposing to severe multiorgan autoimmunity with or without susceptibility to infections (2–4). Cytotoxic T-lymphocyte antigen-4 (CTLA-4) haploinsufficiency is included in this group, since the reduced surface availability of CTLA-4 ultimately results in Treg cell dysfunction (5). The three entities that negatively affect CTLA-4 function in human disease are CTLA-4, lipopolysaccharide-responsive and beige like anchor protein (LRBA), and DEF6 deficiencies (5). These three have been grouped under the term immune checkpoint defects (6). They share common features of dual symptoms of immune dysregulation and immune deficiency, with variable disease expressivity even in individuals with the same mutation (5, 7). Shared phenotypic manifestations of immune dysregulation include autoimmune cytopenia, enteropathy, and lymphoproliferation (5, 8, 9). This triad combination is not commonly seen in other PIRDs (5).

The long-term therapeutic approach for affected patients is challenging (5, 10). Approximately one-third of patients may present mild symptoms and remain stable under supportive care (8). The remaining patients may develop severe multiorgan autoimmunity requiring lifelong immunosuppressive treatment with Treg-sparing immunosuppression [mammalian target of rapamycin (mTOR) inhibitors] or targeted soluble CTLA-4-Ig (abatacept, belatacept) (10, 11). Currently, hematopoietic stem cell transplantation (HSCT) is offered to these patients with treatment-resistant immune dysregulation (1, 12). Nevertheless, little experience in HSCT in these conditions is reported (10), mostly in LRBA deficiency (13). Doubts regarding bridge or remission induction therapy (11, 13, 14), conditioning for the transplant (15), and post-HSCT chimerism goals remain.

Detailed clinical observations can provide insight into the challenges of HSCT management in this subgroup of PIRD patients and more so when they are diagnosed worldwide, including Eastern Europe (5), and the HSCT approach can be variable. Thus, we present case 1 (CTLA-4 haploinsufficiency) and case 2 (CTLA-4 insufficiency-like phenotype), both manifested with autoimmune cytopenia, enteropathy, and lymphoproliferation, with typical lung and central nervous system (CNS) involvement, and variable impairment of humoral responses. Both patients underwent HSCT, and the main complications were mixed chimerism, infections, and immune-mediated complications.

## Clinical description

### Case 1

**Figure 1:**
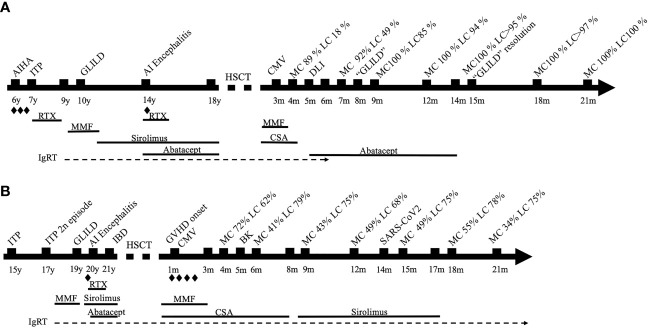
**(A)** Timeline of case 1. **(B)** Timeline of case 2. Steroids; AIHA, autoimmune hemolytic anemia; ITP, immune thrombocytopenic purpura; AI, autoimmune; HSCT, hematopoietic stem cell transplantation; CMV, cytomegalovirus; MC, myeloid chimerism; LC, lymphoid chimerism; CSA, cyclosporin; DLI, donor lymphocyte infusion; GLILD, granulomatous-lymphocytic interstitial lung disease; RTX, rituximab; IgRT, immunoglobulin replacement therapy; IBD, inflammatory bowel disease; GVHD, graft-versus-host disease.

We present an 18-year-old man with CTLA-4 haploinsufficiency [*CTLA4* frameshift mutation c.342_342delC, reported in Schwab et al. (8), subject 42] with low CTLA-4 expression in FoxP3+ CD4 T cell (**Supplementary Figure S1** **)** (16). Patient presented autoimmune and/or inflammatory disorders such as autoimmune hemolytic anemia (AIHA), idiopathic thrombocytopenic purpura (ITP), granulomatous-lymphocytic interstitial lung disease (GLILD), and autoimmune encephalitis, requiring multiple immunosuppressants as shown in **Figure 1**. HSCT was indicated due to partial response of these immune dysregulatory manifestations despite targeted treatment. His immune deficiency and dysregulation activity (IDDA) score (11) prior to HSCT was 17.6. Main characteristics of his baseline disease and HSCT are presented in **Table 1**. The patient received HSCT from his identical human leukocyte antigen (HLA) sister, both sharing blood type and positivity for cytomegalovirus (CMV). The European Group for Blood and Marrow Transplantation (EBMT) guidelines were followed for HSCT using treosulfan, fludarabine, and thiotepa as conditioning treatment and cyclosporin and mycophenolate for graft-versus-host disease (GVHD) prophylaxis (17). The clinical course was complicated with low donor lymphoid chimerism and an episode of lung lesions that posed the differential diagnosis with GLILD. Both resolved with the combination of donor lymphocyte infusions (DLIs) from the identical HLA sister and expert management of immune modulation including abatacept introduction post-HSCT, as described below. The timeline is depicted in **Figure 1**.

**Table 1:** Table 1 Summary of the two cases.

	Case 1-♂	Case 2-♂
Post-HSCT		
Age at disease onset (years)	6	15
Age at CVID diagnosis (years)	10	15
Positive genetic diagnosis	CTLA-4 haploinsufficiency (13 years old)	No
Immune workup at CVID diagnosis		
Absolute lymphocytes (cells/mm^3^)	1,100 (low)	1,200 (low)
CD19+ lymphocytes (%/abs)	13.9/153 (normal/normal)	11/132 (low/low)
CD4+ lymphocytes (%/abs)	43.9/473 (normal/normal)	32/384 (normal/low)
CD8+ lymphocytes (%/abs)	30.8/339 (normal/normal)	31/372 (normal/ normal)
CD19+ naive (IgM+IgD+CD27-) (%, abs)	97.3/149 (high/ normal)	78/102 (normal/normal)
CD19+ switched memory (IgD-CD27+)(%, abs)	0.5/1 (low/low)	12/16 (normal/low)
CD19+CD21^low^CD38^low^ (%,abs)	Not determined	22.8/30 (high/normal)
Naive CD3+CD45RA+ (%)	54 (normal)	35.9 (normal)
Memory CD3+CD45RO+ (%)	31 (normal)	37.9 (normal)
IgG (mg/dl)	588 (low)	576 (low)
IgA (mg/dl)	18 (low)	44 (low)
IgM (mg/dl)	43 (normal)	34 (low)
** **		
Immune workup before HSCT		
Absolute lymphocytes (cells/mm^3^)	900 (low)	700 (low)
CD19+ lymphocytes (%/abs)	22.3/200 (normal/normal)	0/0 (under RTX. low/low)
CD4+ lymphocytes (%/abs)	43.9/395 (normal/normal)	25.4/177 (low/low)
CD8+ lymphocytes (%/abs)	23.5/211 (normal/normal)	44/308 (high/normal)
CD19+ naive (IgM+IgD+CCD27-) (%/abs)	98.5/97 (high/ normal)	NA
CD19+ switched memory (IgD-CD27+)(%/abs)	0.4/0.8 (low/low)	NA
CD19+CD21^low^ CD38^low^ (%)	3.5 (normal)	NA
Naive CD4+ (CD4+ CD45RA+) (%)	52.9 (normal)	17.5 (low)
Eff.mem.CD4+(CD4+CD45RA-CCR7-) (%)	6.6 (normal)	55.4 (high)
Naive CD8+ (CD8+ CD45RA+) (%)	68 (normal)	10.8 (low)
Eff. mem.CD8+ (CD8+CD45RA-CCR7-)(%)	16.3 (normal)	75.5 (high)
IgG (mg/dl)	882 (normal, under IgRT)	1,381 (normal, under IgRT)
IgA (mg/dl)	12 (low)	24 (low)
IgM (mg/dl)	34 (low)	24 (low)
IgRT	Yes	Yes
Infections	CMV chronic infection	Periorbital infection
	Recurrent enteric infections (*Campylobacter* spp. and *Salmonella* spp.)	
Immune-dysregulatory phenotype	Evans syndrome	Immune thrombocytopenia
	Granulomatous-lymphocytic lung disease	Granulomatous-lymphocytic lung disease
	Autoimmune encephalomyelitis	Autoimmune encephalomyelitis
	Lymphoproliferation (splenomegaly, adenomegalies)	Inflammatory bowel disease
		Lymphoproliferation (splenomegaly)
Immunosuppressants prior to HSCT (see **Figure 1**)	Steroids, mycophenolate, sirolimus, rituximab, abatacept	Steroids, mycophenolate, sirolimus, rituximab, abatacept
IDDA score	17.6	21.6
**Post-HSCT**		
Age at HSCT (years)	18	21
Karnofsky	100%	90%
Conditioning regimen	Flu-Treo-Thio	Flu-Treo-Thio
GVHD prophylaxis	Alemtuzumab, CSA, MMF	Alemtuzumab, CSA, MMF
Type of donor	BM-identical HLA MSD	BM-10/10 HLA MUD
	TNC 2.3 × 10e8/kg	TNC 1.63 × 10e8/kg
Graft failure	No	No
Slow engraftment at day 28	Yes	Yes
Infections	CMV, Herpes simplex	CMV, ADV, BK , SARS-CoV-2
GVHD	No	Yes, acute GVHD (CSA switched to sirolimus due to renal disease)
Mixed chimerism 3 months post-HSCT	Yes	Yes
(both lineages)		
Interventions for mixed chimerism	Switch CSA to abatacept	No
	DLI	
Off IS	14 m post-HSCT (abatacept)	17 m post-HSCT (sirolimus)
Off IgRT	Yes, since 6 m post-HSCT	No
Immune workup 21 m post-HSCT		
Absolute lymphocytes (cells/mm^3^)	2,500 (normal)	800 (low)
CD19+ lymphocytes (%/abs)	27/675 (high/high)	4.9/19 (low/low)
CD4+ lymphocytes (%/abs)	25/628 (low/normal)	25/100 (low/low)
CD8+ lymphocytes (%/abs)	36/900 (high/normal)	56/222 (high/low)
NK cells/mm^3^ (%/abs)	8/200 (normal/normal)	8.2/32 (normal/low)
Naive CD4+ (CD4+ CD45RA+) (%/abs)	47.9/300.8 (normal/normal)	30.2/30.2 (low/low)
Eff.mem.CD4+(CD4+CD45RA-CCR7-)(%/abs)	26.8/168.3 (normal/normal)	40.1/40.1 (normal/low)
Naive CD8+ (CD8+ CD45RA+) (%/abs)	21.1/189.9 (normal/normal)	13.7/30.4 (low/low)
Eff.mem.CD8+ (CD8+CD45RA-CCR7-)(%/abs)	2.9/26.1 (low/low)	35.4/78.58 (normal/low)
CD19+ naive (%/ abs)	97.2/656.1 (high/high)	97.4/18.5 (high/low)
CD19+ switched memory (IgD –CD27+)(%/abs)	1.6/10.8 (low/low)	1/0.0 (low/low)
IgG (mg/dl)	1,497 (normal)	870 (normal, under IgRT)
IgA (mg/dl)	<3 (low)	<3 (low)
IgM (mg/dl)	137 (normal)	19 (low)

HSCT, hematopoietic stem cell transplantation; m, months; CVID, common variable immunodeficiency; NA, not applicable; Eff.mem., effector memory; IgRT, immunoglobulin replacement therapy; CMV, cytomegalovirus; IDDA, immune deficiency and dysregulation activity (ref 12); yo, years old; Flu, fludarabine; Treo, treosulfan; Thio, thiotepa; GVHD, graft-versus-host disease; CSA, cyclosporin; MMF, mycophenolate; BM, bone marrow; HLA, human leukocyte antigen; MSD, matched sibling donor; MUD, matched unrelated donor; TNC, total nucleated cell dose; ADV, adenovirus; DLI, donor lymphocyte infusion; IS, immunosuppressant; RTX, rituximab; SARS-CoV-2, severe acute respiratory syndrome coronavirus 2.

Three months post-procedure, split chimerism showed myeloid lineage of 89% and lymphoid lineage of 18% from the donor. In order to improve the chimerism, cyclosporin was quickly withdrawn over 4 weeks and abatacept as a target therapy was started to control the dysregulated lymphocytes that could be left from the recipient. Three weeks later, chimerism remained very low in the lymphoid lineage (18%); therefore, the patient received three DLIs (total dose CD3 1.3 × 10^8^) from his sister with no signs of GVHD after that. Seven months after transplant, 3 months after stopping cyclosporin and switching to abatacept, and 1.5 months after DLI, chimerism improved, showing 92% of myeloid and 49% of lymphoid from the donor.

**Figure 2:**
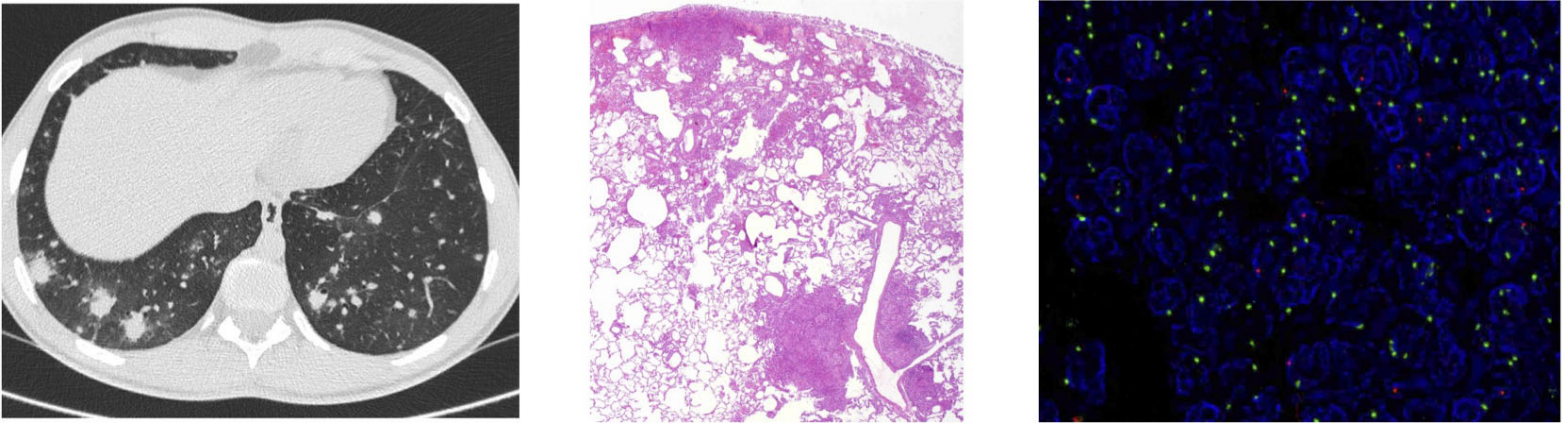
Study of lung lesions of case 1. First image computed tomography (CT) scan showing multiple lung nodules. Second image wedge luyng biopsy demonstrated a non-necrotizing granulomatous inflammation concentrated bronchovasculocentrically and paraseptally [hematoxylin and eosin (H&E), 2x]. Third image fluorescencein-situ hybridization (FISH) using locus specific indentifier (LSI) SRY. In the lymphocytic infiltrates, up to 27% of the nuclei showed a green dot (CEP X) and a red dot (SRY), and 73% of the nuclei showed two green dots (CEP X), thus indicating that the majority of the lymphocytic infiltrate corresponded to the female donor.

Eight months after transplantation, the patient presented fever and diarrhea. Infectious screening was positive for *Campylobacter coli* in a stool culture. After 5 days of fever and on antibiotics, computed tomography (CT) scan showed multiple lung nodules suggesting an infection or lymphoproliferative disorder (**Figure 2**). The study was completed with brain magnetic resonance imaging, positron emission tomography (PET) scan, bronchoalveolar lavage (BAL), and bone marrow aspirate. Multiple adenopathies and splenomegaly were seen. Bone marrow aspirate was normal. Epstein–Barr virus (EBV) was positive in blood and in the BAL, although at a very low number of copies (<250 copies/ml), and the patient did not present other signs of posttransplant lymphoproliferative disorder (PTLD).

A lung wedge biopsy was performed, demonstrating non-necrotizing granulomatous inflammation concentrated bronchovasculocentrically and paraseptally, made up of histiocytes admixed with a lymphocytic component, predominantly T-cell CD4+. Microbiological tests ruled out infection. These findings were suggestive of a GLILD flare (18). To confirm which T cells (female donor vs. male patient) were driving this inflammation, fluorescence *in situ* hybridization (FISH) X/Y was performed in the lung tissue to assess the chimerism *in situ*. The same pattern as in the blood (**Figure 2**) was observed, ruling out enrichment of the patient’s T cells in the granulomas. Lung lesions presented remission within 2 weeks without further intervention. The patient remained on abatacept as the sole immunosuppression.

Fifteen months post-HSCT, the patient presented good clinical evolution with an improvement in the CT scan and respiratory test. Chimerism presented sustained improvement, maintaining 100% and >95% in myeloid and lymphoid lineages, respectively. In this context, abatacept was discontinued.

Currently, 21 months posttransplantation, CT scan and pulmonary function tests are within normal range and the patient’s chimerism is 100% in both lineages. He is off immunosuppression with good immune reconstitution and also off immunoglobulin replacement therapy (IgRT) (**Table 1**).

### Case 2

We present a 21-year-old man with common variable immunodeficiency (CVID) and a phenotype of immune dysregulation similar to CTLA-4 haploinsufficiency characterized by autoimmune cytopenia, inflammatory bowel disease (IBD), lymphoproliferation (GLILD), autoimmune encephalitis, and humoral deficiency requiring multiple immunosuppressants (**Figure 1**). No genetic defect was identified in a gene panel including inborn errors of immunity (IEIs) and PIRD-related genes (SureSelect Custom Constitutional Panel 17 Mb, Agilent), and an array comparative genomic hybridization (aCGH) was normal. The patient fulfilled the European Society for Immunodeficiencies (ESID) Registry Working Definitions for the Clinical Diagnosis for Common Variable Immunodeficiency (19) (**Table 1**). However, CTLA-4 expression in FoxP3+ CD4+ T cells resembled that of CTLA-4-deficient patients, with a marked decrease in the CTLA-4^hi^FoxP3+CD4+ T cells (**Supplementary Figure S1**). HSCT was indicated due to a partial control of immune dysregulation despite sirolimus and abatacept. The IDDA score prior to HSCT was 21.6. The patient received HSCT from an HLA-identical (10/10) unrelated donor with the same blood type. The patient was CMV-positive, while the donor was negative. The clinical course was complicated by GVHD, mixed chimerism, and infectious episodes, as described below. The timeline is depicted in **Figure 1**.

One month post-HSCT, he developed grade 2 GVHD requiring steroids and poor engraftment requiring granulocyte colony-stimulating factor and eltrombopag. He also presented CMV infection requiring preemptive treatment with foscarnet, cidofovir, and specific T-lymphocyte infusion. Thereafter, CMV infection was controlled, but he developed BK hemorrhagic cystitis, community-acquired pneumonia, and a severe acute respiratory syndrome coronavirus 2 (SARS-CoV-2) upper respiratory tract infection.

Mixed chimerism persisted around 60%–70% in the lymphocytic lineage (**Figure 1**). Cyclosporin was switched to sirolimus for the treatment of GVHD at 8 months post-HSCT due to renal dysfunction. Lymphoid chimerism remained in similar range, with a slight increase to 75%. At 17 months post-HSCT, with controlled GVHD, immunosuppression was slowly discontinued with no complications. As the patient maintained a stable lymphoid chimerism above 70%–75% and persistent grade 2 GVHD, no further interventions were performed.

Currently, 21 months post-HSCT, the patient is off immunosuppression. He still presents mixed donor chimerism of 34% in granulocytes and 75% in T lymphocytes without autoimmune episodes. His immune cellular and humoral reconstitution is still incomplete, requiring IgRT and antimicrobial prophylaxis (**Table 1**).

## Discussion

Currently, the management of CTLA-4 haploinsufficiency and similar diseases characterized by marked T-cell activation is still in discussion. Bridge or remission induction therapy (11, 13, 14), transplant conditioning (15), and post-HSCT chimerism goals need to be refined. The two cases reported here are representative of this group of patients who fail to respond to conservative immunosuppressant treatment and move to HSCT with high levels of immune dysregulation. Their post-HSCT outcome and management highlight the importance of an individualized approach to achieve maximum, if not full, lymphoid chimerism to ensure disease remission and complete immune reconstitution.

Over the last decade, allo-HSCT outcomes in IEI have improved significantly. The survival rate for conventional IEI transplants is now approaching 90% (17, 20, 21). HSCT is also being offered to young adults with high rates of success (22). This is due to improved donor selection, better management of HSCT complications, and optimized supportive care (20, 23, 24). Still, high levels of hyperinflammation prior to transplant may promote a greater incidence of alloreactivity disorders post-HSCT (25, 26), and has already been shown to worsen HSCT outcomes, with a greater risk of GVHD (as observed in case 2) and toxicity, and impaired immune reconstitution (25). Also, although the same conditioning and GVHD prophylaxis were given in both cases, they received different types of graft: case 1 received marrow from his HLA-identical sister and case 2 received marrow from a 10/10 HLA unrelated donor. Still, some immune disparities could be present and interfere in the immune reconstitution phase, as described before (25, 27). In addition, patient 2 presented a higher IDDA score and higher number of effector memory CD4+ cells prior to HSCT. All these factors may have contributed to the different post-HSCT outcome in both patients. 

In our cases, HLA-matched donors (one related, one unrelated) and bone marrow source were used. In this sense, it is important to bear in mind that because of the genetic nature of most IEIs, genetic screening of family donors is warranted regardless of symptoms, since PIRDs can display a late or variable clinical onset, as typically described for CTLA-4 haploinsufficiency (8). In order to minimize the higher incidence of alloreactivity, the role of biologic modifiers or targeted therapies as a bridge to HSCT in PIRDs is an important field to explore (14). In our center, both patients received targeted immunosuppression including abatacept until 2 weeks prior to transplantation. Currently, one of the main questions that arise is the dichotomy between lifelong immune modulation vs. HSCT for IEI and PIRD (28).

Current recommendations do not identify patients with CTLA-4 haploinsufficiency who might benefit from long-term targeted immunomodulation vs. HSCT nor the optimal timing for HSCT (10). HSCT outcomes must be balanced with the risks of disease. Reports of HSCT for CTLA-4 haploinsufficiency are scarce. They illustrate that HSCT can be effective (8, 29). Schwab et al. (8) reported 12 transplanted patients among 90 symptomatic *CTLA4* mutation carriers undergoing allo-HSCT between 10 and 50 years of age. Main indications were uncontrollable cytopenia, enteropathy, and lymphoma with additional autoimmune disorders involving lymphoproliferative and infectious complications (8). Nine of the 12 patients (75%) are alive, three of them more than 5 years after HSCT and currently well without medication. Slatter et al. (29) described eight pediatric patients with CTLA-4 haploinsufficiency who underwent HSCT. All received transplants from 10/10 HLA-matched unrelated donors following a reduced-intensity conditioning regimen; the outcome was 50% GVHD, 25% autoimmune disorders, and an overall survival rate of 75% (29). In the report by Chan et al. (24), 13 of the 226 transplanted patients were CTLA-4 haploinsufficiencies, but no specific subgroup data analysis could be performed. The informed nature of decision-making for clinicians, patients, and families in these ill-defined situations is improved if clinical outcome data of defined patients with defined treatments are reported. For this purpose, a systematic description of patients with CTLA-4 haploinsufficiency undergoing HSCT is needed. This description should include IDDA score prior to HSCT. The IDDA score (11) can be used to compare semiquantitative values in one individual over time (clinical course, longitudinally) or between individuals or cohorts at a specific time point (cross-sectionally). The new IDDA version includes broader manifestations of immune dysregulation, factors that indicate the quality of life and need for supportive care, and the occurrence of malignancies (30). This is critical because the absence of ongoing medication and quality of life are important features that are rarely quantified, and these may be better in patients who have undergone transplantation (31–33). Also, a detailed description of immunosuppressants used prior to and post-HSCT and the dynamics of T-cell chimerism are necessary to enable comparisons of therapeutic approaches. In our patients, disease evolution prior to HSCT was 12 and 6 years, respectively; the patients had a high disease burden with IDDA scores >15 and had received multiple courses of immunosuppressants with only partial or transient responses. Despite the non-compelling abovementioned HSCT data, the transplant choice was made by the patients, mainly motivated by chronic disease fatigue and the low quality of life of two active young adults.

More and more adolescents and young adults (AYAs) with IEI are referred for HSCT (22, 34, 35). For these patients, aging leads to early end organ damage, reduced quality of life, and early death (36). Transplant decision is challenging, as they have survived childhood with conservative management, but HSCT needs to be considered before further deterioration. Both of our reported patients have received adult-stage HSCT with reduced toxicity regimen, and the decision to transplant was based on the lack of disease control despite long-term use of two targeted immunomodulators. For newly identified diseases with alternative targeted therapies (14, 37–39) such as CTLA-4 fusion proteins, careful follow-up of different treatment cohorts is necessary to determine the best treatment modalities in the long-term. The safety and efficacy of abatacept for adult patients with CTLA-4 insufficiency or LRBA deficiency are currently being evaluated in a phase 2 clinical trial (ABACHAI) (40).

For some IEIs, a certain level of mixed chimerism is sufficient to improve the patients’ well-being. But is there a minimum level of donor T-cell chimerism required to correct the immune phenotype of PIRD whose immune pathology is T-cell activation? Chimerism was designed to monitor the percentage of the donor cells after the infusion and not to monitor the disease baseline or malignant relapse (41). Full donor T-cell chimerism in LRBA deficiency has been shown to be positively linked to the probability of remission, although data on the relevance of donor chimerism for cure are still limited (11, 13, 42, 43). In many IEIs, stable mixed donor chimerism does not lead to graft rejection, and it may suffice to correct the underlying immunodeficiency (44). However, it has been shown that donor myeloid chimerism is important for long-term immune recovery of T and B lymphocytes and adequate immune function after HSCT (45). In case 2, low mixed myeloid chimerism was a concern, although no DLIs were considered due to the high risk of GVHD and to donor availability. Some data suggest that mixed chimerism can cause persistent autoimmunity or autoinflammation in these patients (46–48). From the report by Slatter et al. (29), six of the eight patients are alive and well with donor chimerism ranging 85%–100%. So, for patients with CTLA-4 haploinsufficiency who present mixed chimerism post-HSCT, it seems reasonable to aim at high, or even full, T-cell chimerism. In cases of no active GVHD, like in case 1, weaning from the immunosuppressant drugs and performing DLI, especially in matched family donors, could be considered. In case 1, abatacept was also started to immunomodulate the patient’s CTLA-4 haploinsufficient lymphocytes. The use of abatacept prior to HSCT is to modulate autologous activated T cells to control the underlying disease. Its use in the posttransplant phase could be beneficial both in controlling the remaining autologous T cells to reduce the risk of disease flare, but also to modulate allo-reactive donor T cells to reduce the risk of GVHD development (49, 50). On the other hand, if the patient presents GVHD, such as case 2, the approach to slowly withdraw the immunosuppressant drug, or even switch to a Treg-sparing regimen such as sirolimus, can be considered. It is difficult to ascertain what the main reasons are for such a different outcome. Our hypothesis is that GVHD development in case 2 [linked to a matched unrelated donor (MUD) and higher levels of inflammation at the time of HSCT] was one of the main determinants in the different management of mixed chimerism and final outcome, since it obliged to a certain level of immunosuppression and interfered with immune reconstitution.

Beyond chimerism, during the early phase of PIRD patient transplant, the recipient’s dysregulated lymphocytes are still a concern, especially in mixed chimerism. Therefore, close monitoring of inflammation and autoimmune complications is crucial for early flare recognition until chimerism and immune reconstitution are complete. In case 1, it is difficult to determine whether the lung nodular lesions were a flare of his GLILD in the context of persistent low chimerism and his being off abatacept (no infection or PTLD was demonstrated, and he was respiratory asymptomatic so suspicion of an immune reconstitution phenomenon was reduced). Also, no CT had been performed in the first 8 months post-HSCT to enable comparisons. The predominance of donor T cells in the nodules along with the improved chimerism thereafter might explain the quick resolution of the lung nodules in a GLILD. In patient 2, currently with 75% of lymphoid chimerism, no disease flare has been observed while off immunosuppression. Immunological biomarkers are still to be defined in the monitoring of PIRD patients’ immune reconstitution and detection of immune dysregulation post-HSCT. Functional studies on the underlying genetic defect (i.e., transendocytosis test for checkpoint deficiencies) might be of interest in cases of persistent mixed lymphoid chimerism (6, 8, 51).

## Conclusion

CTLA-4 haploinsufficiency encompasses a heterogeneous and often complex group of patients requiring an individualized therapeutic approach. Detailed descriptions of case reports and HSCT outcomes are crucial to identify strategies to improve allo-HSCT outcomes and help delimit the target T-cell chimerism and thereby avoid disease flares. These strategies may include the use of targeted immunomodulators not only prior to but also post-HSCT. Furthermore, we need to define post-HSCT-specific monitoring and evaluate improvements of disease burden with specific scores. All of the foregoing is necessary to enrich the informed nature of the decision-making in lifelong management of children and adults with these diseases.

## Patient perspective and informed consent

Written informed consent was obtained from the individuals for the publication of any potentially identifiable images or data included in this article.

## Data availability statement

The original contributions presented in the study are included in the article/**Supplementary Material**. Further inquiries can be directed to the corresponding authors.

## Ethics statement

Written informed consent was obtained from the individual(s) for the publication of any potentially identifiable images or data included in this article.

## Author contributions

AM-S and ÀD-M: These authors share first authorship; AM-S, ÀD-M, and JT: These authors contributed equally to this work; FF-A and LA: These authors share last authorship. All authors contributed to manuscript revision, read, and approved the submitted version.
