# Supplementary figures and images for: Case report: Challenges in immune reconstitution following hematopoietic stem cell transplantation for CTLA-4 insufficiency-like primary immune regulatory disorders
Source: Front Immunol. 2022 Dec 27;13:1070068. doi: 10.3389/fimmu.2022.1070068 (PMC9831655; doi:10.3389/fimmu.2022.1070068)

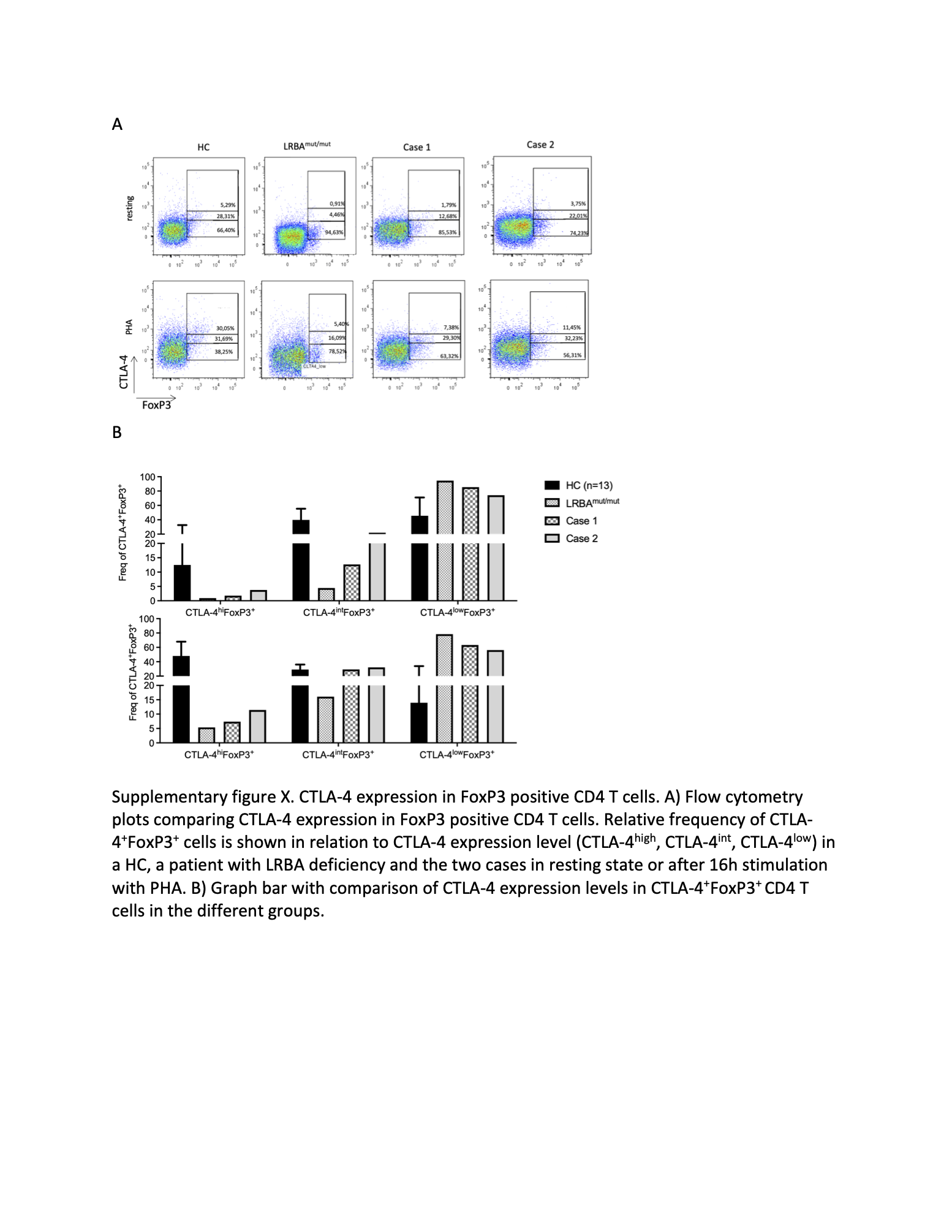

Supplement: Supplementary file 1 [file Image_1.tiff]
